# The Role of Primary Mitochondrial Disorders in Hearing Impairment: An Overview

**DOI:** 10.3390/medicina59030608

**Published:** 2023-03-19

**Authors:** Virginia Fancello, Giuseppe Fancello, Silvia Palma, Daniele Monzani, Elisabetta Genovese, Chiara Bianchini, Andrea Ciorba

**Affiliations:** 1 ENT & Audiology Unit, Department of Neurosciences, University Hospital of Ferrara, 44124 Ferrara, Italy; 2 Department of Otorhinolaryngology, Careggi University Hospital, 50134 Florence, Italy; 3 ENT & Audiology Department, University of Modena and Reggio Emilia, 41100 Modena, Italy; 4 ENT & Audiology Department, University of Verona, 37134 Verona, Italy

**Keywords:** mitochondrial diseases, sensorineural hearing loss, genetics, metabolic disorders

## Abstract

*Background*. Defects of mitochondrial DNA (mtDNA) involved in the function of the mitochondrial electron transport chain can result in primary mitochondrial diseases (PMDs). Various features can influence the phenotypes of different PMDs, with relevant consequences on clinical presentation, including the presence of hearing impairment. This paper aims to describe the hearing loss related to different PMDs, and when possible, their phenotype. *Methods.* A systematic review was performed according to PRISMA guidelines, searching Medline until December 2022. A total of 485 papers were identified, and based on specified criteria, 7 were included in this study. *Results.* A total of 759 patients affected by PMDs and hearing loss were included. The age of patients ranged from 2 days to 78 years old, and the male-to-female ratio was 1.3:1. The percentage of subjects affected by hearing loss was 40.8%, (310/759), and in most cases, hearing impairment was described as sensorineural, bilateral, symmetrical, and progressive, with different presentations depending on age and syndrome severity. *Conclusions*. PMDs are challenging conditions with different clinical phenotypes. Hearing loss, especially when bilateral and progressive, may represent a red flag; its association with other systemic disorders (particularly neuromuscular, ocular, and endocrine) should alert clinicians, and confirmation via genetic testing is mandatory nowadays.

## 1. Introduction

Hearing loss is the most prevalent sensorial deficit, with a major impact on health perception. Depending on the age of onset, hearing loss, if not rehabilitated, has been linked to different disabilities, such as impaired language development in children and impaired interpersonal relationships, academic performances, social lives, and career opportunities, and even to cognitive decline in older adults [1,2]. 

It has been reported that more than 50% of pediatric cases of sensorineural hearing loss (SNHL) have a genetic etiology with a different mode of inheritance: autosomal dominant, autosomal recessive, X-linked, or mitochondrial. Even if most of the hereditary hearing loss is related to disorders of nuclear genes, in the recent literature, a role of mitochondrial genes has been claimed, since also these mutations have been described to be causative of SNHL [3]. 

Mitochondria are sophisticated organelles of human cells, involved in key metabolic processes; they control several metabolic activities, such as oxidative phosphorylation of the electron transport chain [4,5], representing the primary energy source for most biochemical and physiological cellular processes. An imbalance in mitochondrial function can result in their structural damage, which is identifiable as an enlargement and modification of their cristae, with a subsequently decreased membrane potential and ATP synthesis, and the increased production of reactive oxygen species (ROS) and cytochrome C. All of these events can trigger cellular apoptosis and eventually contribute to cellular death [6].

Within the inner ear, mitochondria are strictly related to the preservation of inner ear crucial functions, such as the generation of cochlear endolymphatic potential, as well as the transduction processes driven by hair cells and spiral ganglion neurons. It is evident that any impairment of the delicate energetic equilibrium of inner ear cells may result in the onset of SNHL [3].

Nowadays, the term primary mitochondrial disorders (PMDs) describe, in the literature, a heterogeneous group of conditions related to defects of mitochondrial DNA (mtDNA) and/or of nuclear DNA (nDNA) encoding for structural mitochondrial subunits, causing disorders of the mitochondrial respiratory chain [7,8]. Compared to nDNA, mtDNA mutations are 10–100 times more frequent [1]. Furthermore, different proportions of mutant and wild-type mtDNA might coexist in the same cell, defining a condition called heteroplasmy. The term heteroplasmy refers to not only the coexistence of two or more mitochondrial DNA variants inside different cells in the same subject but also within the same cell or even within a single mitochondrion. The heteroplasmy levels in an individual organ might vary substantially, taking into account that each cell contains thousands of copies of mtDNA [9]. The proportion of normal to mutant mtDNA required for cellular malfunction is known as the “threshold effect” [10]. It is postulated that before an “energetic” failure is observed, often more than 80% of mtDNA in a cell should harbor pathogenic changes [11]. However, this threshold can be different depending on the organ involved [12], since the number of mitochondria per cell varies according to tissue metabolic activity. Therefore, organs with high energy metabolisms are mainly involved in PMDs, and brain and muscular functions are often impaired in such diseases, leading to clinical pictures of encephalomyopathy [13]. Among the inner ear [14], the energetic failure of these organelles can compromise the delicate function of the hair cells or the spiral ganglion neurons [15], thereby leading to sensorineural hearing loss. In Caucasians, it has been reported that about 5% of postlingual deafness can be related to mitochondrial disorders, while the percentage in Asians could be even higher [15]. 

This review aims to describe the sensorineural hearing loss related to different PMDs, and when possible, their phenotypic presentation.

## 2. Materials and Methods

A systematic review of the literature was performed using the Medline bibliographic database to identify relevant peer-reviewed published papers, in order to select the most comprehensive studies focused on hearing loss and mitochondrial disorders. 

The search was performed without any limitation and by two researchers (V.F. and G.F.) from inception to 30 December 2022. The search strategies were developed utilizing Medical Subject Headings (MeSH) terms.

Papers including patients affected by PMDs were identified. 

Keywords used for the search were (“Mitochondrial Diseases” [MeSH]) AND “Hearing Loss” [MeSH]. 

A total of 485 papers were identified in the search.

The inclusion criteria were:

- Studies on humans;
- Studies on PMDs;
- Studies with genetic analysis;
- Original studies on Cohort of patients >20;
- Studies including hearing loss details.

The exclusion criteria were:

- Studies containing duplicated data from other published work;
- A cohort of patients <20;
- Studies published in languages other than English;
- Studies not focused on PMDs;
- Studies not including hearing loss details;
- Studies not including genetic analysis;
- Studies on specific family groups;
- Studies on animals.

Of the papers initially selected, seven met the inclusion criteria. 

**Figure 1:**
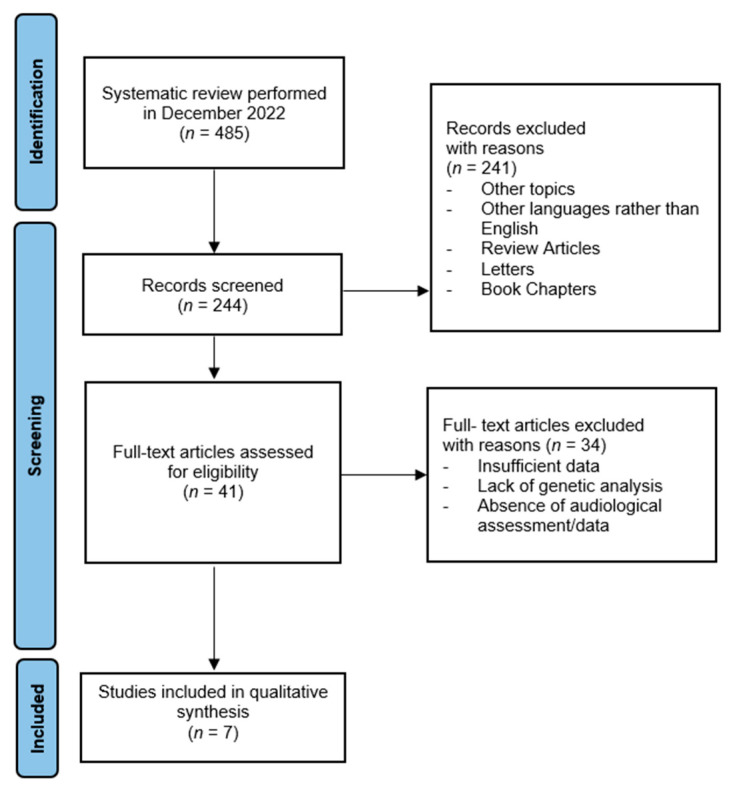
The literature evaluation and selection according to PRISMA criteria [17].

This review was conducted using the preferred reporting items for systematic reviews and meta-analyses (PRISMA) guidelines [16]. The flow diagram is illustrated in Figure 1.

The research was based on previously published studies; thus, no ethical approval or patient consent was required.

Two reviewers worked independently to obtain data. Evidence was extracted from the included studies and entered into an Excel database to collect the following information: first author, year of publication, country, age, sample size, gender, type of DNA defect (mitochondrial or nuclear), percentage of patients who developed SNHL, study design, and the phenotypic manifestation of the genetic alteration.

## 3. Results

The literature search identified seven papers, accounting for a total of 759 patients affected by PMDs who were investigated for hearing loss. All studies except two were retrospective. 

The year of publication ranged from 2006 to late 2022. The age of patients ranged from 2 days to 78 years old, and the male-to-female ratio was 1:1.3. The demographics and characteristics of the selected studies are summarized in Table 1.

In each study, the percentage of subjects affected by hearing loss among a cohort of patients with PMDs was evaluated, and in most cases, the PMDs were sensorineural, bilaterally symmetrical, and progressive (Table 2), with different degrees of severity and age at presentation.

The overall prevalence of SNHL among patients affected by PMDs was 40.8%, (310/759) with high variability among the papers. PMDs can be categorized according to the site of genetic impairments because of defects of mitochondrial DNA (mtDNA) mutations and deletions, or nuclear DNA (nDNA) mutations. 

**Figure 2:**
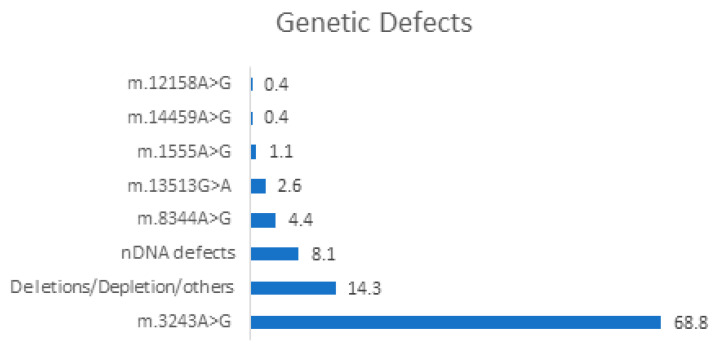
The distribution of PMD different mutations and other gene defects (such as deletion, depletion, and overexpression). All numbers in the figure are expressed as percentages.

It was possible to extract data about the genetic features of SNHL in all except one study, accounting for 272 of 310 patients. The mutation distribution is described in Figure 2. 

Among mtDNA alterations, the most frequently described was m.3243A>G, reported in all of the selected papers and accounting for 68.8% of cases. It was also responsible for a variety of phenotypes, with hearing impairment ranging from mild to profound at the time of the diagnosis, with an estimated mean progression of 5 dB per year by Sakata et al. [9]. The main PMDs and their systemic manifestation are presented in Table 3. 

Usually, in the context of mitochondrial multisystemic disease, SNHL presents as part of a complex clinical picture. Rarely does SNHL manifest as the onset or as the sole sign. Syndromes associated with m.3243A>G were the most frequent, as expected. 

**Figure 3:**
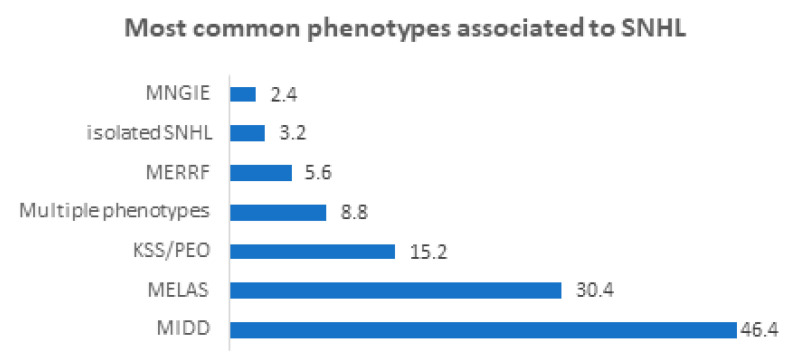
The phenotypes commonly associated with hearing loss in the current review [21,23,24,25]. All numbers in the figure are expressed as percentages.

The most common phenotypes associated with SNHL are illustrated in Figure 3.

Where available, the information regarding the degree of hearing loss was analyzed. Only three papers described this aspect of SNHL, which was accordingly stated as mild in 40%, moderate in 34.7%, and severe to profound in 25.3% [19,20,22].

In some cases, high frequencies seemed to be mainly involved in the early stages of the diseases, displaying a descending slope curve on pure tone audiometry [19,22].

Most of the studies described an SNHL with postlingual onset. However, three papers reported early diagnosis before the age of 6 years old. Specifically, Elander et al. described a cohort of children affected by prelingual deafness and mtDNA impairment, postulating a possible relationship between mtDNA defects and severe hearing involvement. 

Finally, different degrees of hearing loss require different treatment options. In this regard, cochlear implant (CI) was reported by three authors, with a reported total number of 12 patients who underwent surgery, accounting for an overall percentage of 3.8%.

## 4. Discussion

Understanding mitochondrial pathophysiology is a significant challenge for modern medicine, with increasing scientific interest in recent decades. Since the year 2000, an increasing number of papers have been published on this topic. (https://pubmed.ncbi.nlm.nih.gov/?term=%22Mitochondrial+Diseases%22%5BMesh%5D&sort=date&size=50, last accessed on 30 December 2022). 

The mitochondrial genome is mainly involved in encoding different proteins of the electron transport chain and ATP synthesis, key elements for the generation of cellular energy, and the control of cellular apoptosis. Therefore, mutations of mitochondrial DNA can affect cellular metabolism, triggering apoptosis; these features are particularly evident for cells with high energy demands, such as neurons and myocytes. Although there are more than 350 identified pathogenic mutations responsible for mitochondrial disorders, establishing a genotype–phenotype correlation is still difficult [31,32], and consequently, the comprehension of the pathophysiology of PMDs is still challenging. 

Furthermore, the inheritance of mitochondrial disorders differs from Mendelian genetics, and additionally, the mitochondrial genome has a higher variability since it retains (i) a low activity of DNA repair within the mitochondria and (ii) a higher rate of mtDNA mutations due to the continuous exposition to oxygen radicals. In particular, the presence of heteroplasmy (mixed mitochondrial DNA genotypes) has been linked to different mitochondrial diseases (either inherited or acquired) [3,31]. 

The most common mitochondrial disorders linked to hearing loss are mitochondrial encephalopathy, lactate acidosis, and stroke-like episode (MELAS) syndrome; maternally inherited diabetes and deafness (MIDD) [33]; myoclonic epilepsy with ragged red fibers (MERRF) [34], and progressive external ophthalmoplegia (PEO). Furthermore, presbycusis, nonsyndromic hearing loss, or aminoglycoside ototoxicity have been linked to several mitochondrial disorders by some authors [35,36]. 

In 1990, Goto et al. first discovered the m.3243A>G defect in a disorder called MELAS syndrome [37]. Population-based studies suggest that the m.3243A>G is the most common pathogenic mtDNA mutation, with a carrier rate of 1 in 400 people [13]. This specific alteration was present in 68% of patients with SNHL included in this review. According to the literature, the m.3243A>G alters the structure of the mitochondrial leucine tRNA. This modification reduces the activity of the oxidative phosphorylation electron transport chain and increases ROS generation. Consequently, the mitochondrial inner membrane conductance changes, causing a reduction in membrane potential and autophagy and inducing apoptotic cell death [38]. Heteroplasmy plays a crucial role in the pathogenic manifestation of m.3243A>G. According to the literature, the phenotypes linked to m.3243A>G range from asymptomatic to fatal multisystem illnesses. It is observed that subjects with high levels of m.3243A>G mutations manifest a MELAS phenotype, while those with low levels can manifest hearing loss and diabetes [18,20]. Other investigations, though, have shown that hearing loss can develop earlier in patients with higher levels of mutant mitochondrial DNA [39].

Besides MELAS, other syndromes, such as maternally inherited diabetes and deafness (MIDD) [36], myoclonic epilepsy with ragged red fibers (MERRF) [34], and progressive external ophthalmoplegia (PEO) [40] have been related to mitochondrial disorders. In these conditions, SNHL mainly occurs in cases of multiorgan involvement (which often includes the brain, retinal, heart, and endocrine system), such as in MIDD, MERF, and Kearns-Sayre syndrome (KSS) [41]. PEO is hardly associated with severe SNHL, possibly due to the preferential involvement of muscular structures. In addition, the degree of SNHL within the abovementioned diseases is reported to be variable [21]. 

Most of the subjects affected by PMDs and SNHL have been rehabilitated by using hearing aids: the latter represents the main audiological rehabilitative tool for these patients [21]. Interestingly, the first cochlear implant (CI) in a subject affected by profound hearing loss was performed in 1997, in a KSS patient [42]. Since then, various case reports have been published about subjects affected by PMDs and rehabilitated through the use of a CI, with encouraging results in terms of improvements in speech perception and sound detection and, hence, in quality-of-life perception [43]. Considering that, according to histopathology studies, atrophy of the stria vascularis is the most relevant cochlear change in m.3243A>G, while no major changes have been observed in spiral ganglion neurons, electrical stimulation is a feasible rehabilitative option [44,45]. Additionally, in patients with central nervous system degeneration, as observed in MELAS, the central auditory pathway is usually unaffected by the disease, resulting in CI-positive outcomes [46,47]. However, long-term follow-up of implanted patients is required due to the risk of speech perception deterioration, which even if rare, may occur [48]. In addition, CI necessitates in most cases of general anesthesia, and complications during surgery, such as seizures, have been reported [49]. The risk of a cardiovascular event during the surgical procedure should also be considered [50]. Therefore, in the case of a rapid and severe neurodegenerative disease, as observed in mitochondrial neurogastrointestinal encephalomyelopathy (MNGIE) [51], it is necessary to carefully evaluate the natural history and life expectancy before planning a CI. 

In our opinion, the analysis of both nDNA and mtDNA should be included in genetic counseling for SNHL, especially in cases of bilateral progressive postlingual deafness. In particular, when hearing loss is present with other diseases such as diabetes mellitus, especially if diagnosed at a young age in patients with low or normal BMI, maternal family history, and association with other systemic abnormalities, further genetic investigation (including mtDNA testing) should be always performed [27]. 

To understand the inheritance pattern, for genetic counseling, a three-generation family history is necessary, with meticulous attention paid to maternal ancestry [52]. Furthermore, the management of patients with suspected mitochondrial disease should also comprise a complete multisystem clinical review, including cardiac, neurological, endocrinological, and ophthalmic evaluations, as well as long-term monitoring for the development of cardiomyopathy, which can be life-threatening. 

To the best of our knowledge, there are no recognized therapies for PMDs that can modify the course of the disease, thus far [53]. Unfortunately, because of the lack of adequate animal models for mitochondrial disorders, most of the studies on therapeutic strategies remain in the in vitro stage for many years [54,55,56].

The administration of mitochondrial co-factors often referred to as mitochondrial cocktails, such as creatine monohydrate, coenzyme Q-10, arginine, L-carnitine, creatine, and lipoic acid, has been proposed in the literature to (i) slow the progression of PMDs, (ii) increase ATP production, (iii) diminish the use of anaerobic sources, and (iv) decrease the production of reactive oxygen species [57,58]. Unfortunately, the results of such trials have so far been inconsistent since the evidence base for clinical efficacy is limited, and a lot of research still has to be carried out before establishing safe and effective clinical treatments for these conditions to improve patient care [59,60].

Newly reported treatments for PMDs include (i) gene therapy, which is extremely complicated since the expression of mutant mtDNA differs through tissues [61]; and (ii) stem cell therapies. Considering the latter, in vitro studies have demonstrated that some stem cells (i.e., mesenchymal stem cells) could provide healthy mitochondria to replace dysfunctional mitochondria and recover energy metabolism in different types of recipient cells, eventually including the inner ear [62]. 

The transplantation of healthy mitochondria obtained from a donor, named mitochondrial augmentation therapy (MAT), into subjects affected by PMDs has been proposed, and few clinical trials are currently running with encouraging preliminary results [54]. It is likely that in a near future, MAT, as well as other therapies, such as stem cells, could help to preserve or restore hearing in the case of PMDs. Further research, particularly about the intracellular mechanisms and the dynamics of the transplanted mitochondria, is necessary for the future development of these types of treatments [57]. 

The main drawbacks of this review are: (i) the significant heterogeneity of the included studies, and, consequently, (ii) the complexities of the data analysis. Specifically, it was not always possible to classify the degree of SNHL. In the case of severe PMDs, the audiological investigations, and thus the features of hearing loss, were not always well described; however, in our opinion, especially for patients with encephalopathy, additional assessments (eventually including auditory brain-evoked responses) may help to rule out concomitant central processing disorders.

## 5. Conclusions

PMDs are rare conditions, not well known, not well researched thus far, and have different clinical phenotypes. Hearing loss, especially when bilateral and progressive, may represent a red flag; its association with other systemic disorders (particularly neuro-muscular, ophthalmic, and endocrine) should alert the clinician, and confirmation via genetic testing is mandatory. 

Additional research about the mitochondrial mechanisms involved in inner ear cellular death is necessary in order (i) to understand the true prevalence and expression of SNHL in PMDs (including studies with long-term follow-up) and (ii) to identify potential therapeutic targets.

Furthermore, establishing the long-term surveillance of patients with progressive SNHL can improve the diagnostic rate of PMDs and the comprehension of the phenotypes of these disorders.

## Figures and Tables

**Table 1:** Study demographics (R: retrospective, P: prospective, M: male, F: female, and # = number of patients).

Authors	Year	Country	Study	#	Sex
Elander et al. [18]	2022	USA	R	193	102 F, 91 M
Van Kempen et al. [19]	2022	Netherlands	R	62	40 F, 22 M
Sakata et al. [20]	2022	Japan	R	27	11 F, 4 M
Xia et al. [21]	2016	China	R	100	60 F, 40 M
Liu et al. [22]	2014	China	P	73	27 F, 25 M
Scarpelli et al. [23]	2012	Italy	R	60	27 F,33 M
Nesbitt et al. [24]	2012	UK	P	129	79 F, 50 M
Scaglia et al. [25]	2006	USA/Canada	R	115	26 F, 19 M
	2006–2022			759	F:M = 1:1.3

**Table 2:** Prevalence (%) of sensorineural hearing loss (SNHL) in the different cohorts of patients (d: days; m: months; and y: years).

Authors	SNHL %	Mean Age	Defects
Elander et al. [18]	26.9%	14 y (2d—78)	mtDNA mutations and deletions, and nDNA mutations
van Kempen et al. [19]	35.5%	43 y (18—66)	mtDNA mutations
Sakata et al. [20]	100.0%	40 y (22—66)	mtDNA mutations
Xia et al. [21]	21.0%	7 y (4—9)	mtDNA mutations
Liu et al. [22]	67.1%	27 y (7—66)	mtDNA mutations and deletions
Scarpelli et al. [23]	46.7%	45 y (8—73)	mtDNA mutations and deletions
Nesbitt et al. [24]	51.2%	(11m—74)	mtDNA mutations
Scaglia et al. [25]	39.1%	7 y (6m—83)	mtDNA mutations

**Table 3:** The systemic manifestations and genetic correlation of main PMDs. POLG (DNA polymerase subunit gamma), OPA (optic atrophy), TYMP (thymidine phosphorylase), RRM2B (Ribonucleotide-diphosphate reductase subunit M2 B).

Mitochondrial Syndromes	Symptoms	Most Frequent Mutations Reported
Mitochondrial Encephalopathy, Lactacidosis, and Stroke-like episodes (MELAS) [26]	Encephalopathy, myopathy, headache, and focal neurological deficits. Other features are cardiac conduction disorders, diabetes, and chronic fatigue.	mtDNA m.3243A>G (80% of cases) m.3271T>C m.3252A>G m.13513G>A
Maternally Inherited Diabetes and Deafness (MIDD) [27]	Family histories of diabetes and SNHL. Other features are encephalatrophy, cerebellar ataxia, migraine, and stroke.	mtDNA m.3243A>G (>80% of cases)
Kearns-Sayre syndrome (KSS) [28] Progressive External Ophthalmoplegia (PEO)	KSS manifests with progressive external ophthalmoplegia (PEO). The main feature is ptosis but can also present as an overlapping syndrome.	nDNA POLG1 or OPA1 mtDNA m.3243A>G 4977bp deletion
Myoclonic Epilepsy with Ragged red Fibers (MERF) [29]	Progressive myoclonic epilepsy is often associated with ataxia.	mtDNA m. 8344A>G
Mitochondrial Neurogastrointestinal Encephalopathy (MNGIE) [30]	Progressive gastrointestinal dysmotility, cachexia, PEO, neuropathy, and leukoencephalopathy.	nDNA TYMP, POLG, or RRM2B

## Data Availability

Not available.
